# Nanosized zeolites as a perspective material for conductometric biosensors creation

**DOI:** 10.1186/s11671-015-0911-6

**Published:** 2015-05-07

**Authors:** Ivan Kucherenko, Oleksandr Soldatkin, Berna Ozansoy Kasap, Salih Kaan Kirdeciler, Burcu Akata Kurc, Nicole Jaffrezic-Renault, Alexei Soldatkin, Florence Lagarde, Sergei Dzyadevych

**Affiliations:** Laboratory of Biomolecular Electronics, Institute of Molecular Biology and Genetics, National Academy of Sciences of Ukraine, Zabolotnogo Street 150, 03680 Kyiv, Ukraine; Taras Shevchenko National University of Kyiv, Volodymyrska Street, 64, 01601 Kyiv, Ukraine; Institute of Analytical Sciences, 5 rue de la Doua, 69100 Villeurbanne, France; Central Laboratory, Middle East Technical University, Dumlupinar Bulvari, 1, 06800 Ankara, Turkey; Micro and Nanotechnology Department, Middle East Technical University, Dumlupinar Bulvari, 1, 06800 Ankara, Turkey

**Keywords:** Zeolite, Silicalite-1, Mesoporous silica spheres, Enzyme adsorption, Urease, Acetylcholinesterase, Conductometric transducer, Biosensor

## Abstract

In this work, the method of enzyme adsorption on different zeolites and mesoporous silica spheres (MSS) was investigated for the creation of conductometric biosensors. The conductometric transducers consisted of gold interdigitated electrodes were placed on the ceramic support. The transducers were modified with zeolites and MSS, and then the enzymes were adsorbed on the transducer surface. Different methods of zeolite attachment to the transducer surface were used; drop coating with heating to 200°C turned out to be the best one. Nanozeolites beta and L, zeolite L, MSS, and silicalite-1 (80 to 450 nm) were tested as the adsorbents for enzyme urease. The biosensors with all tested particles except zeolite L had good analytical characteristics. Silicalite-1 (450 nm) was also used for adsorption of glucose oxidase, acetylcholinesterase, and butyrylcholinesterase. The glucose and acetylcholine biosensors were successfully created, whereas butyrylcholinesterase was not adsorbed on silicalite-1. The enzyme adsorption on zeolites and MSS is simple, quick, well reproducible, does not require use of toxic compounds, and therefore can be recommended for the development of biosensors when these advantages are especially important.

## Background

Enzyme-based biosensors represent the largest and most successful group of biosensors. They are sensitive, selective, and cheap devices that can be used for environmental, clinical, and industrial purposes [1]. The preparation of enzyme-based biosensors requires the enzyme immobilization on the electrode (transducer) surface. Immobilization conditions directly influence the analytical characteristics of biosensors - sensitivity, reproducibility, selectivity, storage stability, etc. Thus, the improvement of methods of enzyme immobilization is an actual trend in the biosensor development [2,3].

Good immobilization suggests a stable attachment of enzymes to the transducer surface. The enzyme molecules should save their activity after immobilization, and the substrate (target analyte) should have good access to the immobilized enzyme. The achievements of material chemistry provide promising opportunities for the developers of new immobilization methods for the biosensor creation [4].

Zeolites are interesting materials for immobilization of enzymes due to their high surface area and participation in different interactions - hydrophobic, hydrophilic, and electrostatic. On the other hand, zeolites do not contain chemically active groups (until such groups are added deliberately) that can damage enzymes, and, generally, zeolites are low toxic [5]. After synthesis, zeolites can be modified to improve adsorption [6,7]. Adsorption of enzymes on zeolites is a mild method of immobilization, which retains the enzyme activity. Thus, adsorption can be successfully used for immobilization of unstable enzymes. The most significant disadvantage of all adsorption methods is instability and gradual leaking of enzymes into the working solution [8,9]. The information about existing biosensors based on enzymes, adsorbed on zeolites, is presented in [10].

The aim of the current work was to develop a procedure for effective attachment of zeolites to the transducer surface and to evaluate the adsorption of different enzymes onto the modified transducers. We planned to determine an optimal adsorbent by comparison of analytical characteristics of the biosensors created using various adsorbents.

## Methods

### Materials

In the work, the following enzymes were used for biosensor creation: urease (EC 3.5.1.5) from *Canavalia ensiformis*, activity 66.3 U/mg (Fluka, Buchs, Switzerland); glucose oxidase (EC 1.1.3.4) from *Penicillum vitale*, activity 130 U/mg (Diagnosticum, Lviv, Ukraine); acetylcholinesterase (EC 3.1.1.7) from electric eel, activity 426 U/mg (Sigma, Seelze, Germany); and butyrylcholinesterase (EC 3.1.1.8) from equine serum, activity 13 U/mg (Sigma, Seelze, Germany). Glycerol, bovine serum albumin (BSA, fraction V), urea, acetylcholine chloride, and butyrylcholine chloride were from Sigma-Aldrich Chemie (Seelze, Germany). Potassium-phosphate buffer (KH_2_PO_4_-K_2_HPO_4_), NaOH, and glucose were produced by Helicon (Russia). Other inorganic substances were of analytical grade (>98%).

All nanoparticles were synthesized in the Middle-East Technical University (Ankara, Turkey) according to the procedures described below.

### Synthesis of nanoparticles

#### Synthesis of nanozeolite beta

Tetraethoxysilane (TEOS) (98%, Aldrich) was used as a silica source. Aluminum isopropoxide (98%, Aldrich), tetraethylammonium hydroxide (TEAOH) (20 wt% in water, Aldrich), and double-distilled water were used as the other reactants. The molar composition of the gel used for the synthesis of nanozeolite beta was 0.25Al_2_O_3_:25SiO_2_:490H_2_O:9TEAOH. Aging with clear solution was continued under static conditions for 4 h. The crystallization was completed within 17 days under static conditions at 100°C in Teflon-lined autoclaves. The product was separated by centrifugation, washed with distilled water, and dried at 40°C [11]. An approximate particle size of nanozeolite beta was 60 nm.

#### Synthesis of nanozeolite L

The molar composition of the gel used for the synthesis of nanozeolite L was 4TPAOH:25SiO_2_:480H_2_O:100EtOH. First, aluminum powder was dissolved in potassium hydroxide (KOH) solution [12]. Colloidal silica (Ludox HS-40, Dupont, Wilmington, DE, USA) was then added under vigorous stirring, and the gel was stirred at room temperature for 5 min. The crystallization continued for 6 days in Teflon-lined autoclaves under static conditions at 170°C. An approximate particle size of nanozeolite L was 60 nm.

#### Synthesis of 450 nm silicalite-1

The optimized molar composition of the gel used for the synthesis of silicalite-1 is 1TPAOH:4TEOS:350H_2_O. By hydrolyzing TEOS with tetrapropylammonium hydroxide (TPAOH) solution, a clear homogeneous solution was obtained under stirring at room temperature for 6 h. The crystallization occurred at 125°C for 1 day. After the reactions, silicalite-1 was separated by centrifugation. Then the particles were washed with distilled water and dried at 80°C. An approximate particle size of silicalite-1 was 400 to 450 nm.

#### Synthesis of 160 nm silicalite-1

The molar composition of the gel used for the synthesis of silicalite-1 is 4TPAOH:25SiO_2_:480H_2_O:100EtOH. By hydrolyzing TEOS with TPAOH solution, a clear homogeneous solution was obtained under stirring at room temperature for 1 day. The crystallization occurred at 98°C for 20 h. After this, the unreacted material was separated from silicalite-1 by centrifugation. The samples were calcined at 600°C for 10 h in air medium. The average size of the silicalite-1 particle was 160 nm.

#### Synthesis of 80 nm silicalite-1

The molar composition of the silicalite-1 nanocrystal gel is 9TPAOH:25SiO_2_:408H_2_O:100EtOH. The solution was obtained using TEOS, TPAOH, and deionized water and aged at room temperature for 1 day. After hydrothermal treatment at 90°C for 20 h, the product was obtained by centrifugation with deionized water at 20,000 rpm. The product was calcined at 600°C for 10 h in air medium. Finally, the nanoparticles were redispersed in ultrasonic bath with ethanol and then dried at room temperature. The average size of the silicalite-1 particles was 80 nm.

#### Synthesis of mesoporous silica spheres

The molar composition of the gel used for the synthesis of mesoporous silica spheres (MSS) was 1.5Na_2_SiO_3_:1CTABr:361H_2_O:7.4CH_3_COOC_2_H_5_. A clear solution was obtained by dissolving cetyltrimethylammonium bromide (CTABr) followed by sodium metasilicate (Na_2_SiO_3_) in deionized water and quick addition of ethyl acetate (CH_3_COOC_2_H_5_) under stirring. The homogeneous solution was aged at room temperature for 5 h in Teflon-lined autoclave. The hydrothermal treatment proceeded at 90°C for 50 h without stirring. The product obtained was washed in deionized water and ethanol and then filtered and calcined at 600°C for 8 h.

#### Synthesis of zeolite L

The molar composition of the gel used for the synthesis of zeolite L was Al_2_O_3_:20SiO_2_:10.9K_2_O:1030H_2_O. KOH, deionized water, and aluminum sulfate octadecahydrate (Al_2_(SO_4_)3*18 H_2_O) were stirred for 1 h. The second solution containing silica sol (Ludox HS-40) and deionized water was prepared. The final transparent KOH solution was mixed with Ludox solution under vigorous stirring. After 16 h aging with stirring, the solution turned turbid. The hydrothermal treatment proceeded at 180°C for 3 days in Teflon-lined autoclaves. Finally, the products were filtered and washed with deionized water. The calcination temperature was 600°C for 8 h.

### Properties of nanoparticles

Size and morphology of nanoparticles were studied using a scanning electron microscope FEI QUANTA 400F (FEI, Hilsboro, OR, USA). Purity of the samples and properties of the crystals were studied by X-ray powder diffraction (XRD) using Ni-filtered Cu-Kα radiation in Philips PW 1729 (Philips, Amsterdam, The Netherlands). The energy dispersive X-ray spectroscopy (EDX) analyses of all samples were carried out utilizing the Phoenix EDAX X-ray analyzer (EDAX, Mahwah, NJ, USA) equipped with the Sapphire super ultrathin window detector attached to Hitachi S-4700 FE-SEM (Hitachi, Schaumburg, IL, USA).

The Quantachrome Corporation (Boynton Beach, FL, USA) Autosorb-6 analyzer was used for the nitrogen adsorption-desorption experiments. Surface areas of the samples were obtained by multipoint BET, whereas the pore size and pore volumes were obtained by the Saito-Foley (SF) and t-plot methods. The method of samples’ preparation includes their outgassing under vacuum at 300°C for 4 h before analysis.

**Figure 1:**
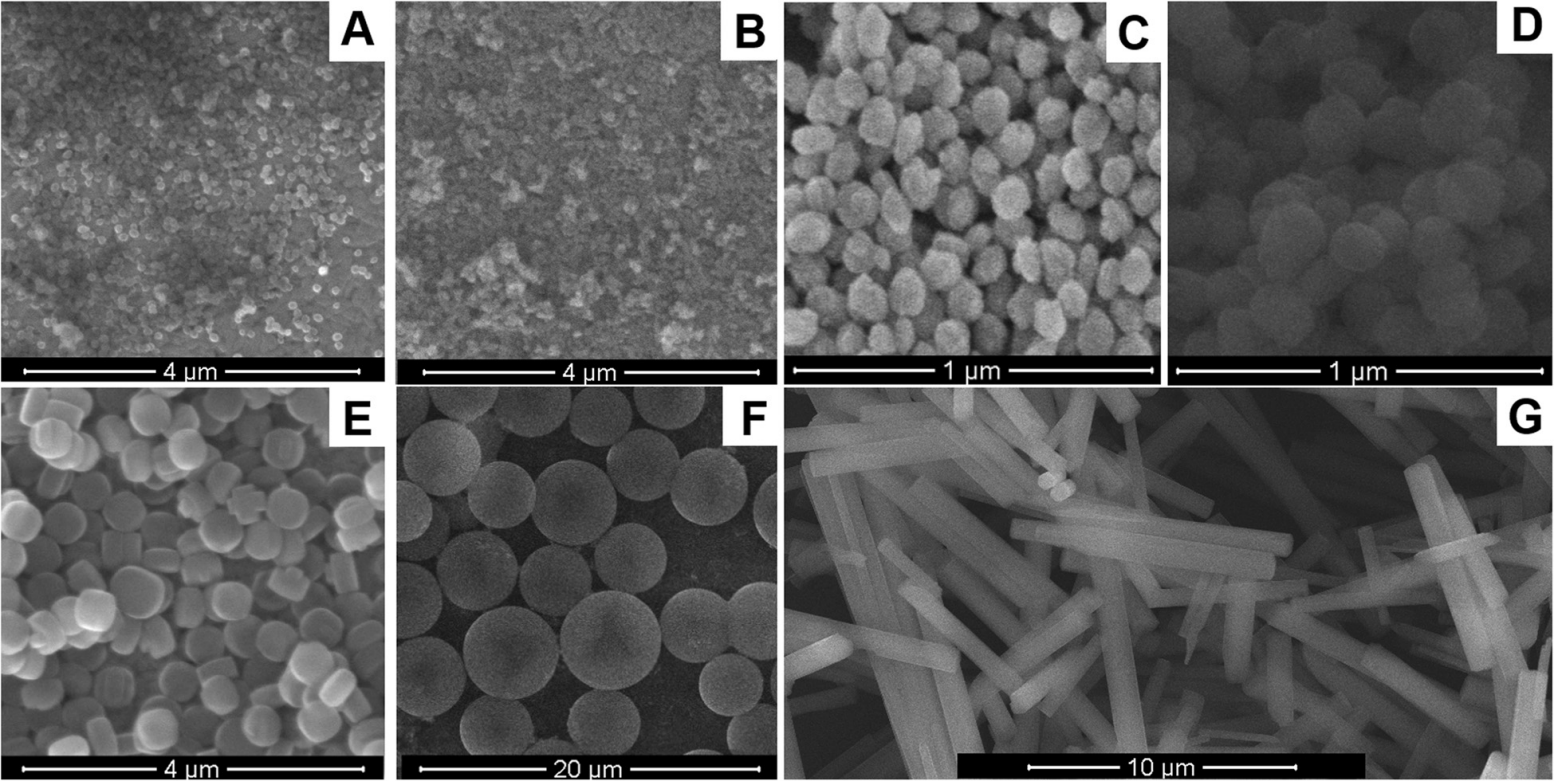
The scanning electron microscopy images of synthesized particles. Nanozeolite beta **(A)**, nanozeolite L **(B)**, 80 nm silicalite-1 **(C)**, 160 nm silicalite-1 **(D)**, 450 nm silicalite-1 **(E)**, mesoporous silica spheres **(F)**, zeolite L **(G)**.

The properties of micro- and nanoparticles are presented in Table 1. SEM images of the zeolites are shown in Figure 1. In our case, the most interesting characteristic of the particles is their surface area. The enzymes used in this work have dimensions 3 to 5 nm; thus, they could not effectively enter the internal pores of the particles (except MSS). As seen, the surface area of most particles was 400 to 500 m^2^/g. However, our biosensors efficiently operated also in the case of silicalite-1 (450 nm) with a smaller surface area.

**Table 1:** **Characteristics of zeolites and MSS**

**Type of particle**	**Average particle size (nm)**	**Surface area (m** ^**2**^ **/g)**	**Pore volume (cc/g)**
Nanozeolite beta	60	472	0.2
Nanozeolite L	60	419	0.18
Silicalite-1 (80 nm)	80	331.9	0.14
Silicalite-1 (160 nm)	160	502.4	0.21
Silicalite-1 (450 nm)	450	281.7	0.56
MSS	4,500	483.5	2.01
Zeolite L	7,000 × 1,100	293.7	0.1198

MSS, mesoporous silica spheres.

The surface of particles was not chemically modified and usually had a negative charge - from −15 to −20 mV (measured by zeta potential at pH 7). Thus, we suggested that the enzymes will interact with negatively charged and hydrophobic parts of the surface (Table 1 and Figure 1).

### Conductometric transducers

Each conductometric transducer consisted of two pairs of interdigitated gold electrodes deposited onto a ceramic support. Transducers were intended to operate in a differential mode of measurements: a biorecognition element (enzymes) was placed on one pair of electrodes, and a reference element (inert protein) on another. The signals were recorded from both pairs of electrodes, and then the signal from reference element was subtracted from the signal from the biorecognition element.

The transducers were manufactured in V. Lashkaryov Institute of Semiconductor Physics of National Academy of Sciences of Ukraine (Kyiv, Ukraine) in accordance with our recommendations. They were 5 mm × 30 mm in size, and the sensitive area of each electrode pair was about 1.0 × 1.5 mm. The width of each digit as well as interdigital space was 20 μm. The photograph and microphotographs of these transducers can be found in [13].

### Modification of transducers with nanosized zeolites

We compared two procedures of attachment of zeolites to the surface of transducers: spin coating with poly(ethyleneimine) (PEI) and drop coating with heating. The second procedure (drop coating) was found to be more effective (see Results and discussion); thus, in most cases, nanoparticles were attached only by drop coating. These procedures did not influence significantly the characteristics of transducers (sometimes the results of differential mode of measurements deteriorated). After experiments, the transducers were cleaned from zeolites with cotton soaked in ethanol.

### Spin coating of transducers

The direct attachment method proposed by Yoon et al. [14] was used to obtain thin layers of zeolites on the surface of transducers. This method includes usage of (PEI) as a linker between zeolite and substrate (in our case, transducer). PEI increased the number of hydrogen bonds between zeolites and substrate and strengthened the interactions between microcrystals and substrates.

First, the surfaces of transducers were dip coated with mucasol (1/6 v/v in distilled water) for 15 min, rinsed with a copious amount of distilled water, and dried under air. Mucasol is an alkaline high-performance universal detergent containing phosphoric acid, tripotassium salt, trihydrate. It was used to change the surface hydrophility and improve the homogeneity of zeolite layers. For formation of homogeneous layers of PEI, both dip coating and spin coating techniques had been tried (the latter was further used since it gave more homogeneous layers). The effects of PEI solvent type (such as hot water and ethanol), PEI concentration (0.5%, 1%, 3%), spin coating time (3000 rpm 15 s, 7 s), and calcination temperature after direct attachment of zeolites (100°C, 90°C, 50°C) were investigated. The obtained zeolite layers were checked using optic microscope. The suitable conditions for zeolite layer production chosen were as follows: spin coating with 0.5% PEI in ethanol at 3,000 rpm for 15 s and calcination at 100°C for 30 min.

### Drop coating of transducers

In case of drop coating, 10% (*w*/*w*) silicalite-1 suspension in 5 mM phosphate buffer, pH 7.4, was used. This suspension was ultrasonicated for at least 20 min, then 0.2 μl of suspension were deposited onto the active zone of each pair of electrodes; afterwards, they were heated at 200°C for 3 min. The procedure resulted in coating transducer surface with an unordered layer of silicalite-1 particles.

### Preparation of bioselective elements

To prepare the biorecognition elements by enzyme adsorption, we used the transducers previously coated with different zeolite particles (see section ‘Modification of transducers with nanosized zeolites’). The same immobilization procedure was used for the transducers prepared by both spin coating and drop coating, and for all enzymes. 0.15 μl of 5% enzyme solution (urease, glucose oxidase, acetylcholinesterase, or butyrylcholinesterase) in 20 mM phosphate buffer, рН 7.4, were deposited onto one pair of electrodes and the same amount of 5% BSA in analogous buffer onto another (reference) pair of electrodes; then, the transducers underwent complete air-drying for 20 min at room temperature. Neither glutaraldehyde nor other auxiliary compounds were used. Next, the transducers were submerged into the working buffer for 10 to 15 min to remove the unbounded enzyme. After the experiments, surfaces of transducers were cleaned from zeolite particles and adsorbed urease using ethanol-wetted cotton.

### Measurement procedure

Conductometric transducers were connected to the portable device for conductometric measurements (9.5 cm × 2.5 cm × 13.5 cm) manufactured in the Institute of Electrodynamics of National Academy of Sciences of Ukraine (Kiev, Ukraine). This device applied sinusoidal potential with a frequency of 36.5 kHz and an amplitude of 14 mV allowed avoiding effects such as faradaic processes, double-layer charging, and polarization of microelectrodes. The nonspecific changes in the output signal induced by the fluctuations of temperature, medium pH, etc. were decreased due to the usage of differential mode of measurement: the conductivity of solution measured by the reference pair of electrodes was subtracted from the conductivity measured by the pair of electrodes with a biorecognition element.

Measurements were carried out at room temperature in continuously stirred 5 mM phosphate buffer solution, pH 6.75, in an open 2 ml cell. The substrate concentrations in the cell were varied by adding different aliquots of the stock solutions (100 mM and 500 mM). All experiments were repeated three times. Each point in the figures corresponds to the average result of three biosensor measurements.

## Results and discussion

### Evaluation of procedures for modification of transducers with zeolites

The first task of the current work was to select an optimal procedure for modification of the transducers with zeolites. The amount of zeolites is very important because, in our case, they serve as carriers of enzymes. On the other hand, too large amount of enzyme on the electrode can impede the diffusion of substances; thus, the biosensor responses will be slower.

**Figure 2:**
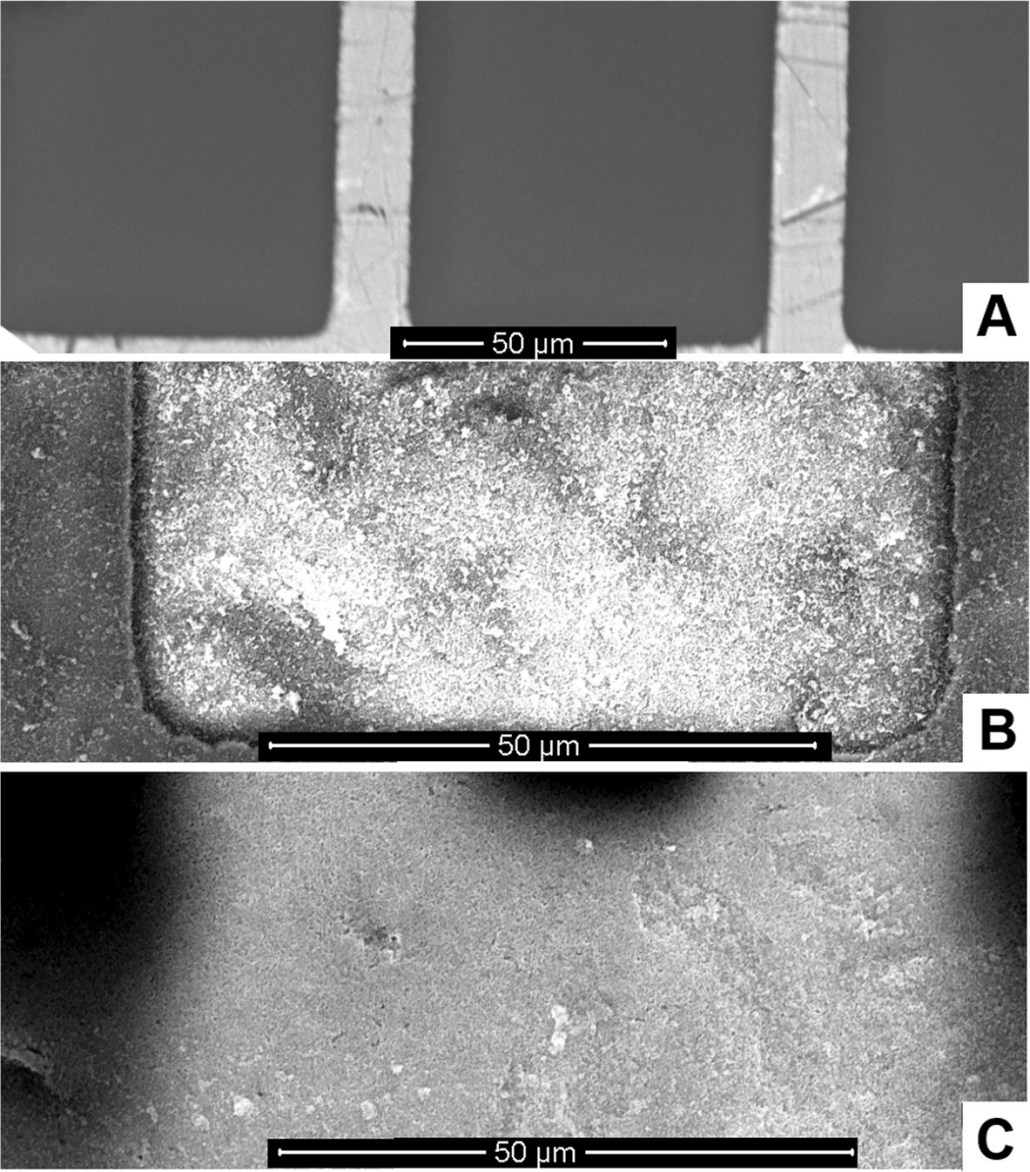
SEM images of conductometric transducers (sensitive parts). Bare surface **(A)**, surfaces covered with silicalite-1 using spin coating **(B)** and drop coating **(C)**.

We used two different procedures for attachment of zeolites: spin coating with PEI and drop coating with heating; the details are in section ‘Modification of transducers with nanosized zeolites.’ The sensitive area of a bare transducer is shown in Figure 2A. Spin coating resulted in the formation of a few zeolite layers (3 to 5) on the surface (Figure 2B). As seen, both parts of the transducer, ceramic (a brighter part of the image) and gold, contained uniform layers of zeolites; however, more zeolites were attached to the ceramic part.

In the case of drop coating, the multiple zeolite layers formed were of different thickness in different parts of the transducer (Figure 2C). Thus, the results of drop coating were not as reproducible as for spin coating. Furthermore, only few zeolites were attached to the gold electrodes (dark part of the image). This can be due to the chemical inertness of gold, whereas the bonding occurred between ceramics and zeolites (Figure 2). An enzyme urease was adsorbed onto the zeolite-modified transducers according to the procedure described in section ‘Preparation of bioselective elements.’ Urease catalyzes urea decomposition due to the reaction:

1 $$ \begin{array}{c}\hfill \kern3.12em \mathrm{Urease}\hfill \\ {}\hfill \mathrm{Urea}+2{\mathrm{H}}_2\mathrm{O}+{\mathrm{H}}^{+}\to 2{{\mathrm{NH}}_4}^{+}+{{\mathrm{H}\mathrm{CO}}_3}^{-}\hfill \end{array} $$

The reaction results in the changes of ion concentrations which leads to local alteration of the conductivity of solution near the sensitive regions of transducer. This allows the usage of conductometric interdigitated electrodes as transducers.

**Figure 3:**
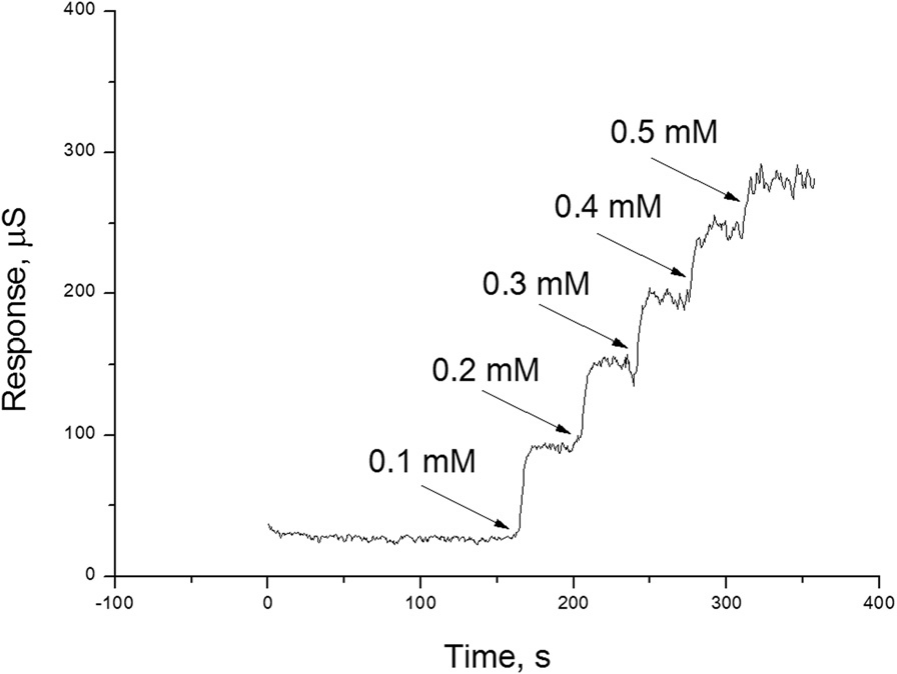
Typical responses of biosensor, based on urease adsorbed on Nanozeolite L, to successive additions of urea (0.1 mM). Concentration of urea (mM) is given on the plot.

The transducers with adsorbed urease can be considered as urea-sensitive biosensors. The analytical characteristics of the obtained biosensors are summarized and compared in Table 2. The table does not show the results obtained for nanozeolites beta and L because they are similar to those obtained for 450 nm silicalite-1 and presented in Table 2. The obtained biosensors exhibited fast response: the signal to urea addition was observed within seconds, and steady-state response was reached in 1 to 2 min. If spin coating was used, the biosensor responses were quicker than in the case of drop coating, which can be explained by a thinner layer of zeolite and a smaller amount of enzyme in the first case. However, such difference in the response time is not significant for biosensor work. Typical responses of the biosensor to several successive additions of urea are shown in Figure 3.

**Table 2:** **Comparison of analytical characteristics of biosensors based on urease adsorbed on silicalite-1-covered transducers**

**Biosensor characteristic**	**Biosensors based on transducers covered via spin coating**	**Biosensors based on transducers covered via drop coating**
Time of steady-state response	1 min	1.5 to 2 min
Sensitivity to urea, μS/mM	700 to 860	1,100 to 1,500
Linear range, μM	10 to 1,000	2 to 700
Limit of urea detection, μM	5 to 10	1 to 2
Reproducibility of responses during working day (RSD)	3% to 8%	2% to 4%
Reproducibility of biosensor preparation (RSD of responses of different biosensors)	12% to 15%	20% to 25%

Generally, the biosensors with transducers modified via drop coating had better characteristics in comparison with spin coating. Furthermore, the drop coating procedure is much simpler. The only significant advantage of spin coating was good reproducibility of morphology of the zeolite layer and, as a result, small difference in the characteristics of different biosensors (RSD of responses <15%). In turn, the biosensors prepared by using the drop coating method were more reproducible (RSD of responses 20% to 25%) than the biosensors based on widespread covalent methods of enzyme immobilization. Thus, the zeolite attachment via drop coating was chosen as an optimal method of the transducer modification.

Furthermore, the experiments with increased amount of silicalite-1 were performed. We repeated the procedure of drop coating for the same transducer two to four times and then adsorbed urease on it. The responses increased by 30% to 40% in the case of double drop coating. The results obtained upon triple/quadruple drop coating did not change compared with double drop coating. Thus, the second drop coating procedure increased amount of silicalite-1 on the surface of transducer (and the amount of adsorbed enzyme), but further repetition was excessive.

### Usage of different silicalites-1, zeolite L, and mesoporous silica spheres for biosensor creation

At the beginning of the work, silicalite-1 (450 nm) and nanozeolites beta and L were tested as adsorbents for creation of urease-based biosensors.

**Figure 4:**
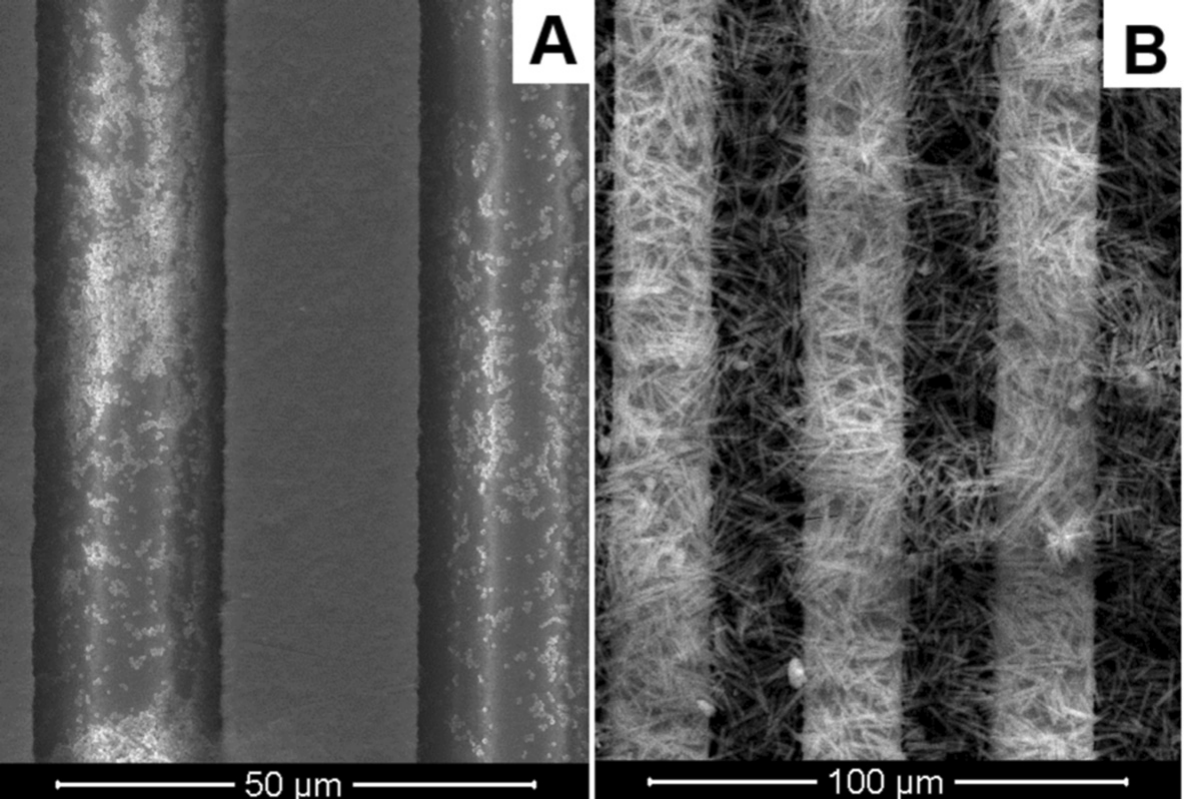
SEM images of conductometric transducers (sensitive parts). The transducers were modified with 160 nm silicalite-1 **(A)** and zeolite L **(B)** via drop coating.

At this stage of work, the urease adsorption on different nanoparticles was studied in order to select an optimal adsorbent for the creation of urease-based biosensor. In the experiments, we used silicalites-1 with dimensions of crystals 80, 160, and 450 nm; zeolite L; and MSS. Properties of the particles are given in Table 1, and photos in Figure 1. SEM images of the transducers covered with 160 nm silicalite-1 and zeolite L are presented in Figure 4.

**Figure 5:**
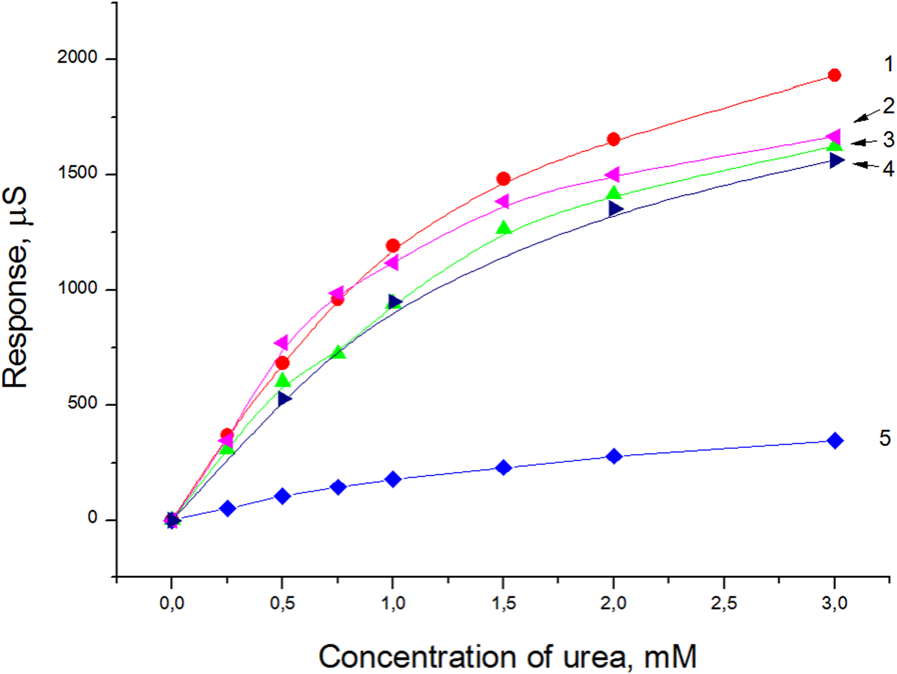
Calibration curves of biosensors based on urease, adsorbed on different particles. Silicalites-1 with dimensions of crystals 450 nm (1), 80 nm (2), 160 nm (4), mesoporous silica spheres (3), and zeolite L (5). Experiments were carried out in 5 mM phosphate buffer, pH 6.75.

Several transducers were modified with each type of the particle, and then urease was adsorbed according to the procedure described in ‘Preparation of bioselective elements.’ After this, the calibration curves for urea determination were obtained, and an averaged curve was calculated. The calibration curves for biosensors with different adsorbents are shown in Figure 5. As seen, the shape of the calibration curves and linear ranges of urea determination were similar in all cases except the biosensor with zeolite L. Unfortunately, direct relation between the size of silicalite-1 crystal and the value of biosensor response was not found. The best responses exhibited the biosensor with 450 nm silicalite-1; the values of responses of biosensors with 80 nm and 160 nm silicalite-1 and as well as with MSS were smaller. However, the biosensors had good characteristics; thus, all mentioned particles can be used for the biosensor creation. The biosensors with zeolite L demonstrated the worst results.

Electron microscopy showed that the amount of MSS on the surface of transducers was smaller than the amount of silicalites (probably MSS did not stick well to the transducer surface), but the biosensor responses were still high. We suggest that the effect of a smaller amount of MSS was compensated by larger pores in the MSS crystals (18 nm); the enzyme molecules could enter these pores, and thus, the effective surface area of MSS was larger than the area of silicalites.

Reproducibility of biosensor preparation was investigated for all particles. Relative standard deviation of responses of different biosensors was 20% to 25%. These results coincide with previously obtained results of reproducibility of the biosensors based on nanozeolites L and beta and demonstrate that the formation of zeolite layers is a quite reproducible process (Figure 5).

### Creation of biosensors based on other enzymes

Finally, it was important to evaluate silicalite-1 (450 nm) as an adsorbent for the creation of other enzyme biosensors. This zeolite was chosen because it showed the best results when developing urease-based biosensors in the previous part of the work.

Currently, dozens of enzymes are used in biosensors. Glucose oxidase (GOD), acetylcholinesterase (AChE), and butyrylcholinesterase (BuChE) are widely used for creation of conductometric biosensors for determination of saccharides, acetylcholine, and different toxic substances [15]. The chemical reactions catalyzed by these enzymes are the following:

2 $$ \begin{array}{c}\hfill \kern0.6em \mathrm{GOD}\hfill \\ {}\hfill \mathrm{D}\hbox{-} \mathrm{glucose}+{\mathrm{O}}_2\to \mathrm{D}\hbox{-} \mathrm{gluconic}\;\mathrm{acid}+{\mathrm{H}}_2{\mathrm{O}}_2\hfill \\ {}\hfill \kern2.64em \downarrow \hfill \\ {}\hfill \kern2.76em \mathrm{acid}\;\mathrm{residue}+{\mathrm{H}}^{+}\hfill \end{array} $$

3 $$ \begin{array}{c}\hfill \kern4.08em \mathrm{AChE}\hfill \\ {}\hfill \mathrm{Acetylcholine}+{\mathrm{H}}_2\mathrm{O}\to \mathrm{choline}+{\mathrm{CH}}_3-{\mathrm{COO}}^{-}+{\mathrm{H}}^{+}\hfill \end{array} $$

4 $$ \begin{array}{c}\hfill \kern4.2em \mathrm{BuChE}\hfill \\ {}\hfill \mathrm{Butyrylcholine}+{\mathrm{H}}_2\mathrm{O}\to \mathrm{choline}+\mathrm{butyrate}+{\mathrm{H}}^{+}1\hfill \end{array} $$

These reactions result in the changes of solution conductivity that can be registered by the conductometric transducer.

**Figure 6:**
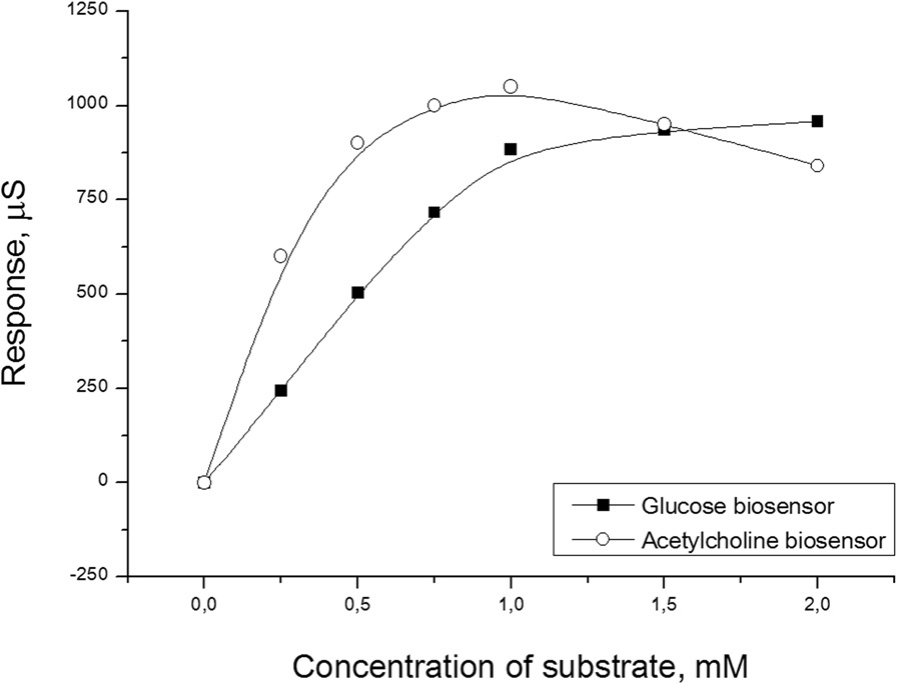
Calibration curves of biosensors based on glucose oxidase and acetylcholinesterase for determination of glucose and acetylcholine. Measurements were carried out in 5 mM phosphate buffer, pH 6.75.

We used the same procedure of enzyme adsorption as in the case of urease. The GOD- and AChE-based biosensors were successfully created. The typical calibration curves for determination of glucose and acetylcholine are shown in Figure 6.

The biosensors based on adsorbed BuChE did not exhibit any response to butyrylcholine. This could be caused either by low BuChE activity or by insufficient adsorption of BuChE on silicalite-1. To check the BuChE activity, we immobilized BuChE using covalent cross-linking between BuChE and BSA via glutaraldehyde; in this case, efficient biosensors were obtained. Thus, we concluded that poor BuChE adsorption on silicalite-1 was the cause of failure.

In the case of adsorption of urease on zeolites, good reproducibility of biosensor preparation was observed, and for this reason, reproducibility was also studied for the GOD- and AChE-based biosensors. Relative standard deviation of responses of different biosensors was 25% (for both glucose and acetylcholine biosensors).

Therefore, we suggest that the development of zeolite-modified biosensors based on adsorbed enzymes is a promising direction of further research.

## Conclusions

In this work, we used nanozeolites beta and L, MSS, and silicalites-1 (80 to 450 nm) for adsorption of the enzyme urease to create urea-sensitive biosensors. Analytical characteristics of the obtained biosensors were compared. Different methods of zeolite attachment to the transducer surface were used. Biosensors with all tested particles except zeolite L were suitable for work. The best characteristics demonstrated the biosensors based on urease adsorbed on transducers modified with silicalite-1 (450 nm) via drop coating. The method of enzyme adsorption on zeolites can be characterized by advantages such as quickness, simplicity, the absence of toxic chemical reagents, and good reproducibility. Furthermore, GOD, AChE, and BuChE were adsorbed on the transducers with 450 nm silicalite-1. Glucose and acetylcholine biosensors were successfully created; BuChE appeared to be unsuitable. Generally, using enzyme adsorption on zeolites can be recommended for the development of biosensors when reproducibility of their preparation and simplicity of immobilization method are especially important.

## References

[1] Wang J (2006). Analytical electrochemistry.

[2] Kimmel DW, LeBlanc G, Meschievitz ME, Cliffel DE (2012). Electrochemical sensors and biosensors. Anal Chem.

[3] Teles FRR, Fonseca LP (2008). Applications of polymers for biomolecule immobilization in electrochemical biosensors. Mater Sci Eng C.

[4] Xu Z, Chen X, Dong S (2006). Electrochemical biosensors based on advanced bioimmobilization matrices. TrAC.

[5] Li Z, Barnes JC, Bosoy A, Fraser Stodart J, Zink JI (2012). Mesoporous silica nanoparticles in biomedical applications. Chem Soc Rev.

[6] Yiu HHP, Wright PA, Botting NP (2001). Enzyme immobilisation using SBA-15 mesoporous molecular sieves with functionalised surfaces. J Mol Catal B: Enzymatic.

[7] Liu AM, Hidajat K, Kawi S, Zhao DY (2000). A new class of hybrid mesoporous materials with functionalized organic monolayers for selective adsorption of heavy metal ions. Chem Commun.

[8] Tsai MC, Tsai YC (2009). Adsorption of glucose oxidase at platinum-multiwalled carbon nanotube-alumina-coated silica nanocomposite for amperometric glucose biosensor. Sensor Actuat B.

[9] Bayramoğlu G, Yalçin E, Arıca MY (2005). Immobilization of urease via adsorption onto l-histidine–Ni(II) complexed poly(HEMA-MAH) microspheres: preparation and characterization. Process Biochem.

[10] Hasanzadeh M, Shadjou N, Eskandani M, Guardia M (2012). Mesoporous silica-based materials for use in electrochemical enzyme nanobiosensors. Trends Anal Chem.

[11] Prokešová P, Mintova S, Čejka J, Bein T (2003). Preparation of nanosized micro/mesoporous composites via simultaneous synthesis of Beta/MCM-48 phases. Microporous Mesoporous Mater.

[12] Larlus O, Valtchev VP (2004). Crystal morphology control of LTL-type zeolite crystals. Chem Mater.

[13] Soldatkin OO, Peshkova VM, Saiapina OY, Kucherenko IS, Dudchenko OY, Melnyk VG (2013). Development of conductometric biosensor array for simultaneous determination of maltose, lactose, sucrose and glucose. Talanta.

[14] Lee JS, Kim JH, Lee YJ, Jeong NC, Yoon KB (2007). Manual assembly of microcrystal monolayers on substrates. Angew Chem Int Ed.

[15] Soldatkin AP, Dzyadevych SV, Korpan YI, Sergeyeva TA, Arkhypova VN, Biloivan OA (2013). Biosensors. A quarter of a century of R&D experience. Biopolym Cell.

